# Improvements to Robotics-Inspired Conformational Sampling in Rosetta

**DOI:** 10.1371/journal.pone.0063090

**Published:** 2013-05-21

**Authors:** Amelie Stein, Tanja Kortemme

**Affiliations:** California Institute for Quantitative Biomedical Research and Department of Bioengineering and Therapeutic Sciences, University of California San Francisco, San Francisco, California, United States of America; University of Michigan, United States of America

## Abstract

To accurately predict protein conformations in atomic detail, a computational method must be capable of sampling models sufficiently close to the native structure. All-atom sampling is difficult because of the vast number of possible conformations and extremely rugged energy landscapes. Here, we test three sampling strategies to address these difficulties: conformational diversification, intensification of torsion and omega-angle sampling and parameter annealing. We evaluate these strategies in the context of the robotics-based kinematic closure (KIC) method for local conformational sampling in Rosetta on an established benchmark set of 45 12-residue protein segments without regular secondary structure. We quantify performance as the fraction of sub-Angstrom models generated. While improvements with individual strategies are only modest, the combination of intensification and annealing strategies into a new “next-generation KIC” method yields a four-fold increase over standard KIC in the median percentage of sub-Angstrom models across the dataset. Such improvements enable progress on more difficult problems, as demonstrated on longer segments, several of which could not be accurately remodeled with previous methods. Given its improved sampling capability, next-generation KIC should allow advances in other applications such as local conformational remodeling of multiple segments simultaneously, flexible backbone sequence design, and development of more accurate energy functions.

## Introduction

Predicting the structures of proteins in atomic detail is one of the key challenges in computational molecular biology. There are two basic difficulties: advancing sampling strategies to more efficiently explore the vast space of possible conformations, and improving energy functions to be able to consistently distinguish correct, native-like from incorrect structures. Sampling and scoring methods are developed under the general assumption that native-like conformations are in the global energy minimum [1]. There are both important exceptions to this rule [2] and cases where a given energy function used for modeling is not able to identify native-like conformations as lowest-energy predictions [3], [4]. However, in the majority of cases native-like conformations indeed have lower energies than non-native models [1]; then sampling conformations close to the native structure becomes the primary bottleneck [5].

Sampling methods need to improve on (i) efficiently searching the vast conformational space, (ii) descending into native-like minima in the rugged energy landscape, and (iii) traversing energy barriers between minima. Strategies for diversification during sampling help increase coverage of conformational space and identify regions in which low-scoring conformations may be found. Such strategies have successfully been applied in initial stages of protein modeling protocols, when multiple different conformations are collected for further exploration [6],[7]. Intensification strategies are instrumental for sampling low-energy conformations in the extremely rugged all-atom energy landscape, where even small deviations from the native structure can lead to drastic energy penalties [5], [8]. Many protein modeling protocols have an initial exploration stage, and the lowest-energy intermediates are then refined in multiple independent simulations in an intensified search for native-like minima [7], [9]. Narrow minima present an additional challenge, particularly in local sampling and high-resolution refinement, where only few conformations are compatible with already tightly packed protein environments. Moreover, different minima may be separated by large energy barriers. These barriers must be traversed to reach near-native conformations from intermediate steps during sampling [10]. Annealing can help traverse such energy barriers by temporarily smoothing the energy landscape and lowering barriers. Annealing strategies have long been used successfully in molecular modeling, with the most well-known application using changes in the simulation temperature over trajectories (simulated annealing) [11], .

In this work we test several strategies for diversification, intensification and annealing in the context of conformational sampling of local regions in proteins, an important yet relatively tractable problem in high-resolution protein modeling in rugged energy landscapes. We show that a combination of intensification and annealing strategies synergistically increases the fraction of near-native protein models generated by robotics-inspired sampling [4] in the protein modeling and design program Rosetta [13]. Given the general nature of these strategies, we believe that many of our conclusions are valid for other applications of robotics-based sampling as well. In particular, the demonstrated dramatic improvements in sampling conformations with sub-Angstrom accuracy for local protein regions make these promising strategies for use in design of new protein conformations and functions [14], [15], [16], [17], [18].

## Results

### Rationale and Overall Strategy

**Figure 1:**
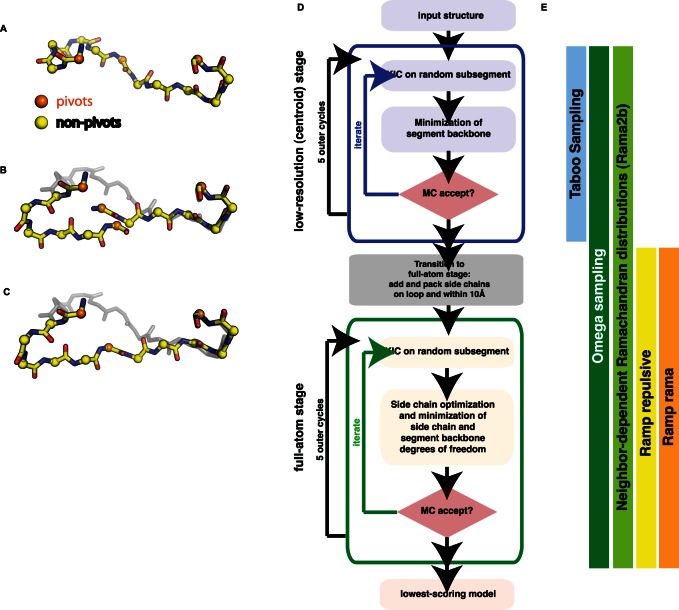
Overview of the Rosetta Kinematic Closure Protocol and New Sampling Strategies Tested Here. Kinematic closure for local conformational sampling: (A) 3 Cα atoms in the segment to be remodeled are designated as pivots (orange), the remaining *N*-3 Cα atoms are non-pivots (yellow). (B) Torsion angles at the non-pivot atoms are sampled from residue-specific Ramachandran distributions in standard KIC [4], opening the segment. (C) Analytical closure calculates values for the pivot φ/ψ torsions that form a closed conformation (“KIC move”). Molecular representations were rendered with PyMol [49]. (D) The Rosetta kinematic closure Monte Carlo Minimization protocol illustrating the low resolution (centroid) and full-atom stages. A KIC move is followed by side chain optimization in the full-atom stage, and by minimization in both stages. A trial conformation is accepted or rejected using the Monte Carlo Metropolis criterion [4]. Iterations in both centroid and full-atom stages are grouped into inner and outer loops. The lowest-scoring model from each such trajectory is reported. (E) The sampling improvement strategies tested here are active during different stages of the Rosetta kinematic closure protocol: Taboo sampling serves diversification and is used in the low-resolution stage to improve coverage of the conformational space by rapidly testing many different conformations with the centroid energy function that simplifies side chain details. The intensification strategies Omega sampling and Rama2b are applied in all stages. Ramping component terms of the energy function as annealing strategies is only used in the high-resolution stage, to overcome large energy barriers in the rugged Rosetta full-atom energy landscape.

We implemented and assessed diversification, intensification and annealing strategies within the Rosetta kinematic closure (KIC) protocol, a robotics-based method that calculates mechanically accessible protein conformations of local protein segments (Figure 1) [4]. A KIC move distinguishes “pivot” and “non-pivot” atoms in the protein segment to be modeled (Figure 1A). Torsion degrees of freedom at non-pivot atoms are sampled according to Ramachandran probabilities, which effectively opens the chain and explores diverse conformations (Figure 1B). Analytical closure then calculates six φ /ψ torsion degrees of freedom at the middle and two outer pivot Cα atoms to exactly close the chain. This step ensures a purely local conformational change only in the chosen segment without affecting the rest of the structure (Figure 1C). KIC moves are then combined with side-chain optimization (“repacking”) and minimization steps into a Monte-Carlo minimization protocol (Figure 1D). This method has previously been shown to successfully sample and identify sub-Angstrom protein conformations for many cases [4].

Here we assess the changes in sampling sub-Angstrom conformations achieved by the different strategies (Figure 1E). The first strategy we tested employs an initial diversification stage (“Taboo” sampling) to increase coverage of conformational space. Taboo sampling records previously chosen backbone conformations and requires future moves to sample different regions of conformational space. The second strategy aims to intensify sampling of certain regions by sampling of neighbor-dependent φ/ψ combinations (“Rama2b” sampling) or of ω angles (“Omega” sampling). The third strategy uses annealing methods that gradually ramp the weight of terms in the Rosetta energy function to overcome energy barriers (“Ramp repulsive” or “Ramp rama” sampling). The diversification and intensification strategies tested here change non-pivot sampling, whereas annealing approaches modulate the energy function.

We evaluated the performance of these strategies on an established benchmark set of 45 12-residue protein segments ([19], [20], Methods). In each case, the segment is deleted from the protein structure and then “reconstructed” *de novo*; all side chains within 10 Å of the segment are modeled at the same time from a standard rotamer library not including the native conformations. Previous methods [19], [20], [21], including standard KIC sampling [4], demonstrated considerable success in sampling and correctly identifying near-native conformations on this benchmark, suggesting that modeling 12-residue segments is a challenging but relatively tractable problem. However, in almost half of the benchmark cases, sub-Angstrom conformations were either not sampled or not identified correctly by the energy function. This benchmark therefore represents a difficult test where improvements over current state-of-the-art approaches require non-trivial advances.

We used two metrics to quantify performance of our tested strategies: the root mean square deviation (RMSD) of the backbone atoms in the remodeled segment between the lowest-scoring model and the native conformation, using the median value across the 45 benchmark cases, and the median percentage of final models (trajectory endpoints) with <1 Å RMSD to the native (in the following denoted as “sub-Angstrom”). The first metric is commonly used and thus provides a comparison to previous work. The latter measure allows observation of changes in the distribution of models towards sub-Angstrom conformations, even if these conformations cannot be distinguished by the current energy function as more native-like. For method development purposes, the relative computational tractability of simulations on 12-residue segments allowed us to test a number of individual and combined sampling strategies and present a side-by-side comparison of different approaches.

### Implementation and Effects of Individual Sampling Approaches

**Figure 2:**
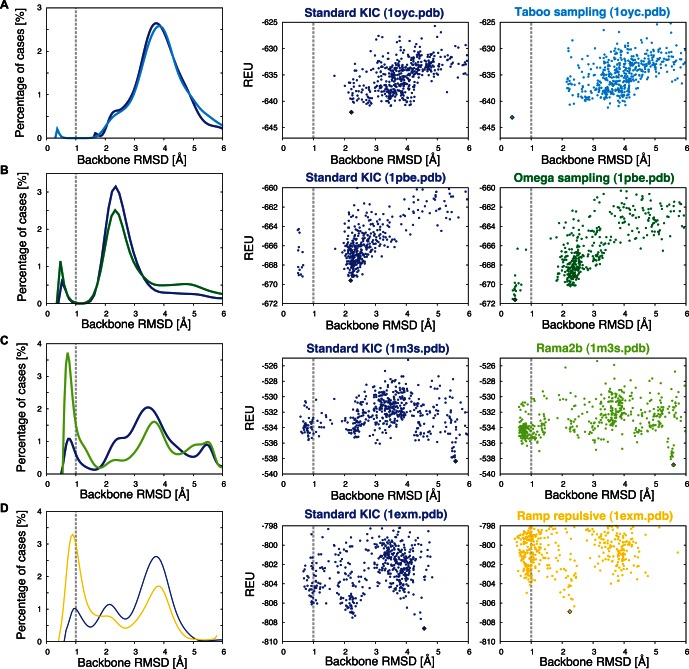
Examples of Improved Sampling by Individual Strategies. RMSD distributions of selected examples for standard KIC (dark blue) and individual sampling approaches tested here (left), along with energy-vs-RMSD plots for standard KIC (center) and individual strategies as described in this work (right). Energies are given in Rosetta energy units (REU). 500 models (dots) were generated for each simulation. The lowest-energy model is indicated by a diamond. (A) Taboo sampling (light blue) on PDB 1oyc identifies a sub-Angstrom model not found by standard KIC. (B) Omega sampling (dark green) on PDB 1pbe enables correct identification of sub-Angstrom conformations by Rosetta energy. (C) Rama2b (bright green) on PDB 1 m3 s and (D) Ramp Repulsive (yellow) shift the distribution towards more native-like conformations.

We first tested Taboo sampling as a strategy for diversification of sampled conformations. Our implementation of Taboo sampling was inspired by recent work on sampling bottlenecks in protein structure prediction that described “linchpin” features. These features, such as specific backbone torsion bins and secondary structure elements, were only rarely sampled in random trajectories but almost always led to low-scoring near-native conformations when they were found [22]. Taboo sampling aims to diversify proposed conformations such that linchpin features are more likely to be sampled. As in [22], we divide Ramachandran space into four bins that roughly correspond to preferred regions of different secondary structure types (Methods). For each proposed conformation, a torsion bin vector is recorded that represents the φ/ψ values for each residue in the modeled segment. To ensure that different conformations are tested, a list of distinct torsion bin vectors then prescribes the bins the non-pivot torsions must be sampled from (Figure 1A–C). When the list of torsion bin vectors to be tested next is exhausted and needs to be updated, the probability of each torsion bin at each position, based on how likely it is given the respective amino acid, is adjusted proportionally to how often the bin has been sampled so far (Methods). Torsion bin vectors that have already been considered are not tested again. This strategy promotes sampling of combinations of multiple less likely torsion bins that may be missed in history-independent sampling. As a result, Taboo sampling may be able to reach conformations that are not sampled by standard KIC despite having a low score (Figure 2A), although finding these rare conformations may not be robust to sampling variations (see also discussion below).

We next implemented and tested two intensification approaches, (i) sampling of ω degrees of freedom (Omega) to explore deviations from planar peptide bonds [23] and (ii) selection of φ/ψ torsion angle combinations from neighbor-dependent Ramachandran distributions (Rama2b) to consider the identities of adjacent residues in sampling [24]. Figure 2B illustrates a case where Omega sampling not only increases the fraction of sub-Angstrom models (left), but also correctly discriminates sub-Angstrom conformations from another set of models (see secondary “funnel” at 2 Å RMSD in the energy landscape, right) that scored similarly in standard KIC sampling (center). This discrimination is remarkable because the added variations in ω do not change the Rosetta energy function; instead, improved sampling is able to generate lower-scoring, more native-like conformations. In contrast, Figure 2C illustrates a case where KIC with Rama2b sampling shifts the distribution of models significantly towards native-like conformations, but these conformations still score worse than other non-native models. Here, clustering analysis would reveal the sub-Angstrom conformation as one of the most represented and thus likely correct conformations (Figure S1). Clustering is commonly used in *ab initio* structure prediction [25], where the coarse-grained energy function is known to be less accurate in discriminating near-native from other models, and has also been applied after high-resolution modeling [5], [26], [27].

As a third strategy, we tested the effect of annealing approaches already used in a number of existing Rosetta applications (e.g. [28], [29]). Here we ramp the weight of fa_rep (the repulsive component of the Lennard-Jones potential in Rosetta, "Ramp repulsive"), as well as the rama score (the likelihood of a φ/ψ combination given the amino acid type, "Ramp rama"), over the course of the outer cycles in the full-atom stage of the KIC protocol (Figure 1D). Annealing allows clashes or unfavorable torsions in remodeled conformations in initial stages of the protocol. This strategy makes it possible to traverse energy barriers that may otherwise be hard to cross. Figure 2D illustrates an example where annealing shifts the population of models towards the native conformation.

### Overall Performance of Individual and Combined Sampling Approaches

**Figure 3:**
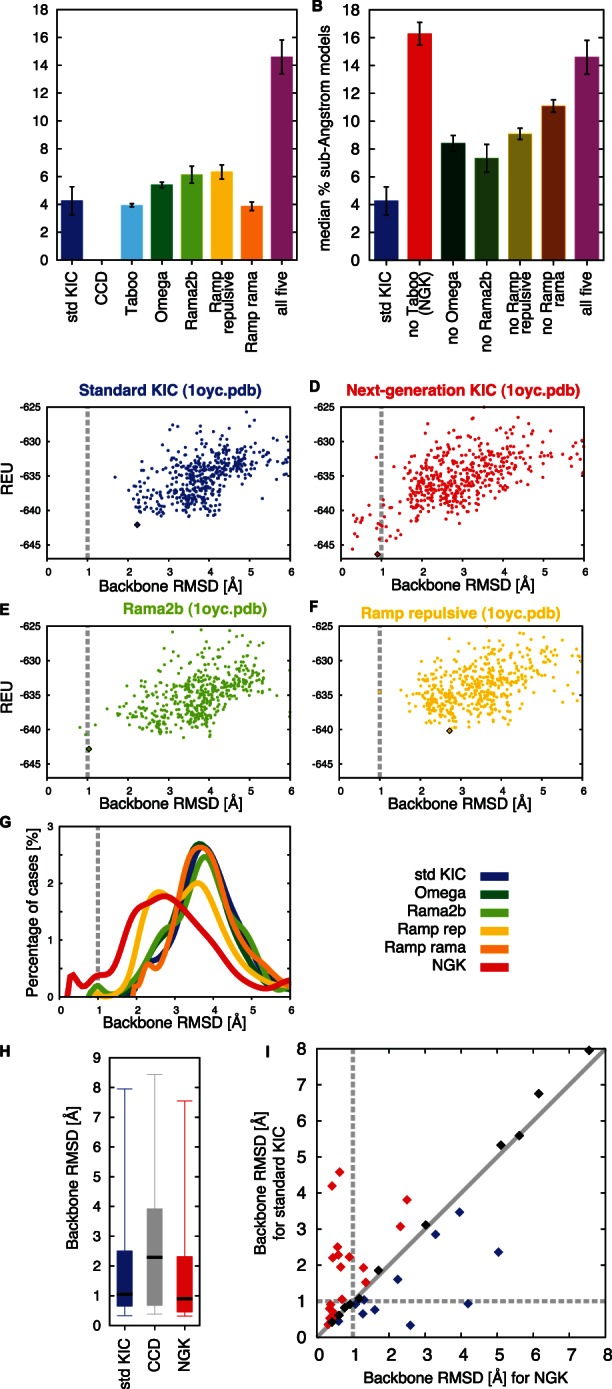
Median Performance Across the 12-Residue Benchmark Sets and Illustration of Synergy. (A) Barplot showing median percent sub-Angstrom (m%sA) across benchmark sets 1 and 2 for the individual sampling strategies tested here as well as their combination (“all five”), showing a clearly increased percentage of sub-Angstrom models. Error bars are standard deviations from 3 independent simulations (generating 500 models for each of the cases in the dataset, repeated three times). Colors are as in Figure 2. The value for CCD is 0 with this measure. (B) Barplots of “leave-one-out” trials in which all combinations of 4 sampling improvement methods are tested: without Taboo sampling (red, NGK), without Omega sampling (dark green), without Rama2b (green), without Ramp repulsive (dark yellow), and without Ramp rama (dark orange). These data are also provided in Table 1. (C–F) Energy-vs-RMSD plots of remodeling PDB 1oyc with standard KIC (C), next-generation KIC (D), Rama2b sampling (E) and Ramp repulsive sampling (F). REU, Rosetta-energy units. (G) RMSD distributions for the different methods. Colors are as in Figure 2, NGK in red. Rama2b and Ramp repulsive sampling both contribute to enabling sampling of sub-Angstrom conformations of the remodeled segment (in 1oyc.pdb), while the other individual strategies do not change the RMSD distribution for this case. Nevertheless, the combined performance in NGK is higher than expected from the individual improvements, indicating synergy. (H) Boxplots of median RMSDs for standard KIC (blue), CCD (gray) and NGK (red), based on the lowest-energy model for each benchmark case. Boxplots show the minimum and maximum among the lowest-scoring RMSDs across the benchmark set (error bars), the 25^th^ and 75^th^ percentile (box boundaries) as well as the median (thick line). Both kinematic-closure-based methods have lower median RMSDs than CCD. (I) Comparison of the lowest-scoring RMSD for each benchmark case in simulations with standard KIC vs. with NGK. NGK typically achieves lower RMSDs than standard KIC (red), while for some cases KIC achieves lower RMSDs (blue). Cases with an RMSD change <10% are shown in black.

In spite of the improvements of the described sampling approaches shown in Figure 2 for selected examples (as measured by increases in how often sub-Angstrom conformations are sampled), their overall effect when applied individually to the entire benchmark set of 45 12-residue segments was only modest (Figure 3A). Individual methods yielded a median value of 3.9–6.3% sub-Angstrom conformations, compared to 4.3% for standard KIC (Figure 3, Table 1). In particular, Taboo sampling and Ramp rama overall did not perform significantly better than standard KIC; Omega, Rama2b and Ramp repulsive sampling consistently improved the fraction of sub-Angstrom conformations sampled over standard KIC (median across the dataset), but only by a small margin.

**Table 1:** Median Percentage of sub-Angstrom Models for Individual and Combined Methods.

Method	avg(m%sA)	stddev	Method	avg(m%sA)	stddev
**standard KIC**	4.3	1.0			
**CCD**	0.0	0.0			
**Taboo sampling**	3.9	0.1	**no Taboo sampling**	16.3	0.8
**Omega sampling**	5.4	0.2	**no Omega sampling**	8.4	0.6
**Rama2b sampling**	6.1	0.6	**no Rama2b sampling**	7.3	1.0
**Ramp repulsive**	6.3	0.5	**no Ramp repulsive**	9.1	0.4
**Ramp rama**	3.9	0.3	**no Ramp rama**	11.1	0.4
**all five**	14.6	1.2			

Average and standard deviation of the median percentage of sub-Angstrom models across the 12-residue benchmark set, calculated from three independent simulations with 500 models for each benchmark case in each simulation. This data is visualized in Figure 3A+B.

However, combining all five methods showed a dramatic improvement in sampling sub-Angstrom models (Figure 3A, magenta bar), reaching a median value of 14.6% sub-Angstrom models. This behavior appears to be due to synergistic effects of the independent improvements, as the fraction of sub-Angstrom conformations is higher than expected from addition of the individual improvements. Error bars in Fig. 3 indicate the variation between three independent simulation sets (each containing 500 decoys for each of the 45 12-residue segments). The observed variation is small compared to the overall increase seen upon combining the different sampling methods.

We next asked whether each of the individual methods contributes favorably to the increased sampling rate of sub-Angstrom conformations by the “all five” method (Figure 3B) by testing all possible combinations of four methods in a “leave-one-out” analysis. Without Omega sampling, Rama2b sampling or either of the annealing strategies, the percentage of sampled sub-Angstrom conformations clearly decreased. In contrast, leaving out Taboo sampling increased sub-Angstrom sampling (Figure 3B, red bar). The negative effect of Taboo sampling on overall performance may be due to the fact that enforcing diversification could bias against native-like conformations that would otherwise be easy to identify in the current energy landscape. It is also likely that the increased coverage of conformational space achieved with the current parameters simply is not sufficient for a stable increase in performance. The latter might be alleviated by longer simulations with increased numbers of attempted Monte-Carlo moves or by communication between different trajectories sampling the same segment; such communication strategies have been successfully applied in protein structure prediction [7], [9].

### Performance of an Improved Combined Protocol: “Next-generation KIC”

Given the results of the “leave-one-out” analysis above, we combined four of the described sampling strategies (excluding Taboo sampling) into an improved protocol, termed “next-generation KIC” (NGK). NGK reached an overall median value of sub-Angstrom conformations sampled in the 45 12-residue segment set of 16.3% (Figure 3B). There is substantial synergy between the different sampling approaches, as adding the individual improvements over standard KIC only yields an expected overall median value of 8.6±2.7% sampled sub-Angstrom conformations (see Table 1 and Methods). The synergy is also illustrated in an example in Figure 3C–G. Comparison of NGK (Figure 3D) to standard KIC (Figure 3C), the effect of selected individual approaches (Figure 3E,F) and the RMSD distributions from each individual method (Figure 3G) show that different strategies contribute to successful identification of sub-Angstrom conformations, either by shifting the overall distribution of models or by enabling sampling of rare but low-scoring conformations. Moreover, the sub-Angstrom median RMSD across the benchmark dataset (Figure 3H) shows that, for the majority of the cases, the lowest-scoring conformation is very close to the native. NGK tends to generate models closer to the native structure than standard KIC (Figure 3I) and it has a slightly shifted distribution of the RMSD percentiles (boxes in Figure 3H; see also Table S1 for RMSDs of lowest-scoring models for all methods). Although we only measure backbone RMSDs, remodeled conformations with sub-Angstrom RMSDs usually also have highly accurate side chain placements, as discussed further below.

**Figure 4:**
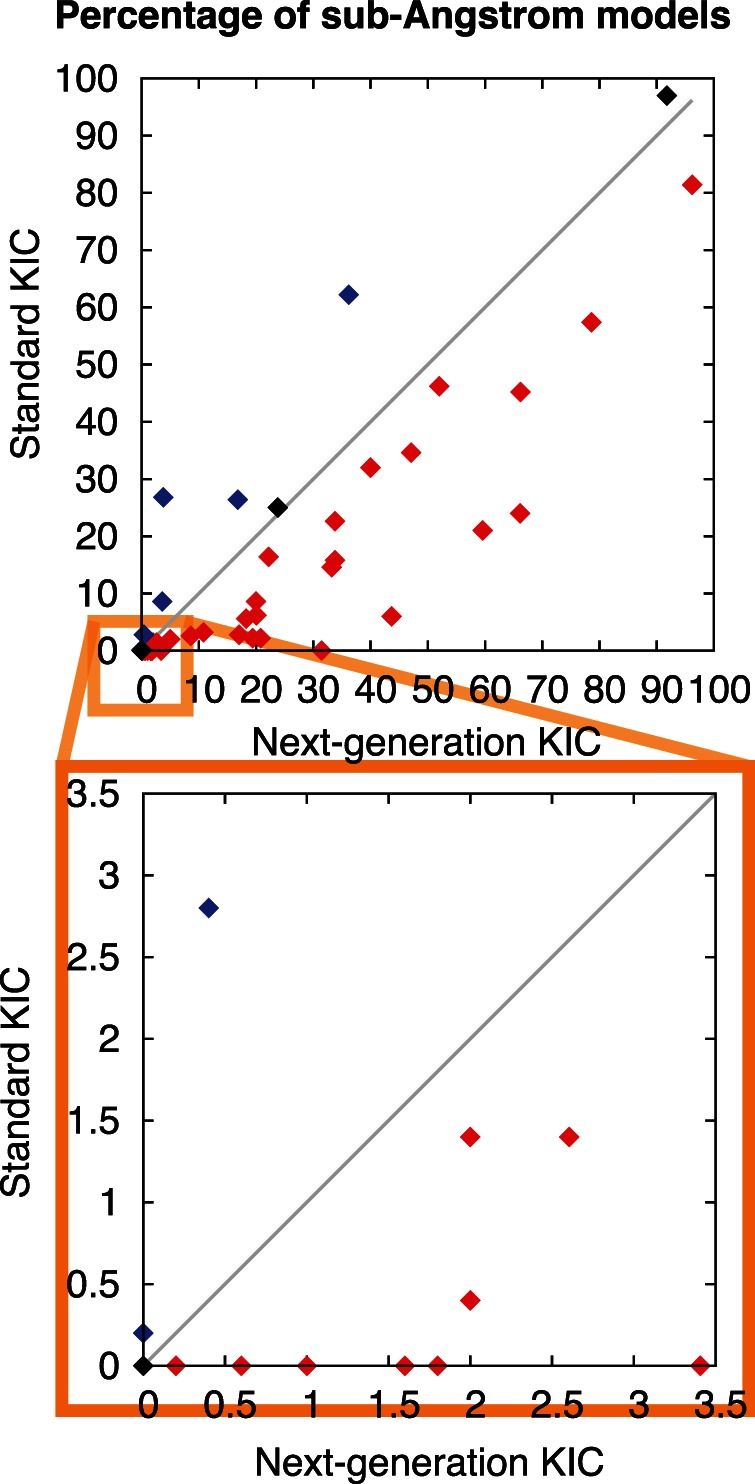
Comparison of the Percentage of sub-Angstrom Models Generated by KIC and NGK. Direct comparison of the percentage of sub-Angstrom models (%sA) between standard KIC and NGK for each of the 45 benchmark cases, grouped into those that are better sampled with NGK (red), those that are better sampled with standard KIC (blue), and those for which %sA did not change much (<±10%, black). Cases that were not at all or very rarely sampled by standard KIC but are more often and consistently found by NGK are specifically highlighted (orange box and bottom panel). %sA and the rank of the lowest-scoring sub-Angstrom model for each individual benchmark case are also given in Figure 5.

**Figure 5:**
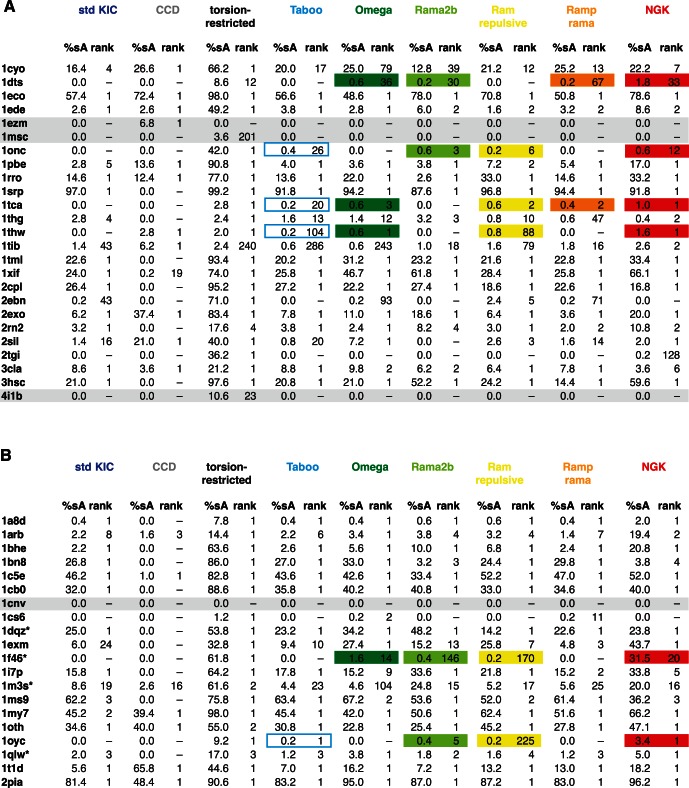
Performance of the different sampling strategies for the 12-residue benchmark set. Performance on all benchmark cases in datasets 1 (A) and 2 (B) is shown in terms of percent sub-Angstrom models (%sA). In addition, the rank of the lowest-scoring sub-Angstrom (<1 Å RMSD) model in a set of 500 is given; rank 1 indicates that the lowest-scoring model has a sub-Angstrom RMSD. As reference performance of existing methods, standard KIC [4] and CCD [20] are also shown. Data for both methods were regenerated using the same Rosetta revision as for all other methods (Methods). Torsion-restricted sampling serves as an additional control. Benchmark cases that do not generate high-ranking (< rank 20) sub-Angstrom conformations either with NGK or in torsion-restricted sampling are grayed out. For six cases where sub-Angstrom conformations were only sampled with NGK, but not standard KIC, the individual sampling strategies that help each particular case are highlighted. Due to synergy between the different sampling strategies, the %sA of NGK often is higher than expected from the individual methods. * indicates that the starting structure is a dimer, with residues in the dimerization interface within 10 Å of the remodeled segment.

Across the benchmark set, the distribution of the fraction of sampled sub-Angstrom conformations is broad: for some structures many models are close to the native conformation, while for other cases the fraction of the sampled conformations that are close to the native is small (Figures 4 and 5). Remarkably, with the combination of intensification and annealing strategies in NGK, increases in the percentage of sub-Angstrom conformations are not only observed for individual instances as illustrated in Figures 2 and 3, but for the vast majority of benchmark cases when comparing to standard KIC (Figure 4).

In addition to these considerable improvements, NGK successfully generated sub-Angstrom conformations that were not sampled at all by standard KIC for six of the 12-residue segments (Figure 4 inset, Figure 5). For each of these cases, several of the new methods individually enable sampling of sub-Angstrom models, often with synergy in the combination (highlighted cases in Figure 5), as also discussed above. Improvements in these rarely sampled cases are especially important, as they allow more reliable identification of sub-Angstrom conformations even for cases in which sampling correct conformations is clearly limiting.

### Analysis of Failures where no Sub-Angstrom Conformations are Sampled by NGK

Despite considerably improved sampling in many of the benchmark cases, NGK still failed to generate sub-Angstrom models for six of the 45 structures in the benchmark (Figure 5). To analyze these “sampling failure” cases, we developed a variant of KIC that restricts sampling of non-pivot φ/ψ combinations to the native torsion bin (defined as in Taboo sampling) for each residue. This “torsion-restricted” method tests whether sub-Angstrom conformations could be generated under much simpler conditions, where sampling is intensified considerably in the vicinity of the native regions. These regions may be difficult to find otherwise because of rarely sampled linchpin features [22].

Torsion-restricted sampling generated low-energy sub-Angstrom conformations for only two of the six failure cases, 1cs6 and 2ebn. Both contain a cis-proline in the remodeled segment. The frequency with which these – generally very rare – conformations are sampled may be too low in the current implementation (Methods), and increasing it may improve identification of sub-Angstrom conformations for these structures.

In two other of the six failure cases, 1msc and 4i1b, sub-Angstrom conformations were generated in torsion-restricted sampling, but these are not the lowest-scoring models. Moreover, when we relaxed the local segment starting from the native structure (“relaxed-natives”, see Methods), we found that these conformations also score worse than non-native conformations sampled by NGK. This behavior generally indicates energy function deficiencies. In addition, analysis with MolProbity [30] showed that both segments contain strained bond angle conformations that may not be covered by the bond angle variation NGK explores in the current implementation (Table S1). KIC and NGK currently only introduce small variations in backbone bond angles, 2.48° around the mean, as observed in high-resolution structures (Methods). We also performed KIC and NGK simulations in which all bond angles are idealized, which gave similar results to sampling with small variations (Figure S2). However, others [31], [32], [33] have successfully used larger variations, which may be beneficial in cases of strained conformations.

In the remaining two cases without sub-Angstrom conformations sampled by NGK (1cnv, 1ezm), even torsion-restricted sampling did not generate sub-Angstrom models. Relaxed-native 1cnv segments score worse than the non-native conformations identified by NGK, indicating energy function deficiencies, as above. In contrast, for 1ezm the relaxed-native segment conformation has a lower energy, indicating a sampling problem (i.e. native-like conformations could have been identified by energy if they had been sampled). MolProbity analysis shows that the remodeled segment contains two bond angle outliers, which may not be covered by the currently implemented bond angle variation, as discussed above (see also Table S1, Methods).

### Analysis of Failures where Sub-Angstrom Conformations are Sampled, but not Correctly Identified: Sampling Improvements Reveal Energy Function Deficiencies

For 15 of the 45 benchmark cases, although sub-Angstrom conformations were sampled, these were not correctly identified by the energy function, i.e. they were not the lowest scoring models generated in the simulation (Figure 5). For six of these “identification failures”, the relaxed-native segment scores lower than the lowest-scoring NGK model; this behavior indicates that sub-Angstrom conformations should be identifiable with further sampling improvements (Table S1). Torsion-restricted sampling identifies low-scoring sub-Angstrom models for these cases (Figure 5), supporting the hypothesis that these can be reached with sufficient sampling. In contrast, the nine other identification failures may reflect energy function deficiencies, as the relaxed-native structure score worse than the NGK models (Table S1).

**Figure 6:**
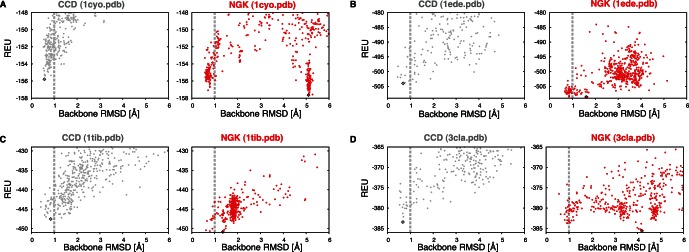
Energy Function Deficiencies Revealed by Sampling Improvements. Energy-vs-RMSD plots of the four benchmark cases for which NGK (red) finds alternative, lower-energy conformations far from the native conformation that were not observed when sampling with the CCD method in Rosetta (gray) [20]: 1cyo (A), 1ede (B), 1tib (C) and 3cla (D). REU, Rosetta-energy units.

A complicating factor in any simplified “sampling” versus “scoring problem” analysis, as above, is that scoring problems may have been masked by less thorough sampling with previous methods. This behavior can be seen in a comparison between simulation results of NGK and another well-established method for local conformational sampling in Rosetta, fragment insertion followed by cyclic coordinate decent (CCD) [20], [34]. CCD relies on fragments extracted from PDB structures [35] and thus performs well whenever sufficiently close fragments are available (here, to ensure suitability of the benchmark for reconstruction of segments in proteins of unknown structure, fragments from homologs are excluded). Overall, CCD samples sub-Angstrom conformations for fewer cases in the benchmark set, leading to a median performance below that of KIC and NGK (Figures 3 and 5, Table 1). Because CCD and KIC use the same energy function and similar numbers of Monte-Carlo evaluation steps in Rosetta, we attribute this difference to the improved sampling afforded by KIC [4]. In all but one of our benchmark cases, whenever CCD identifies sub-Angstrom models, NGK samples these sub-Angstrom conformations as well (Figure 5). However, in four of these 18 cases, NGK generates even lower-scoring but non-native conformations, revealing problems in the Rosetta energy function that were not detected previously by CCD or standard KIC (Figure 6). This suggests that sampling improvements can expose shortcomings of the energy function used to rank modeled conformations. Similar scoring problems have also been observed with other recent sampling improvements [36].

### Reconstructing Longer Segments

**Figure 7:**
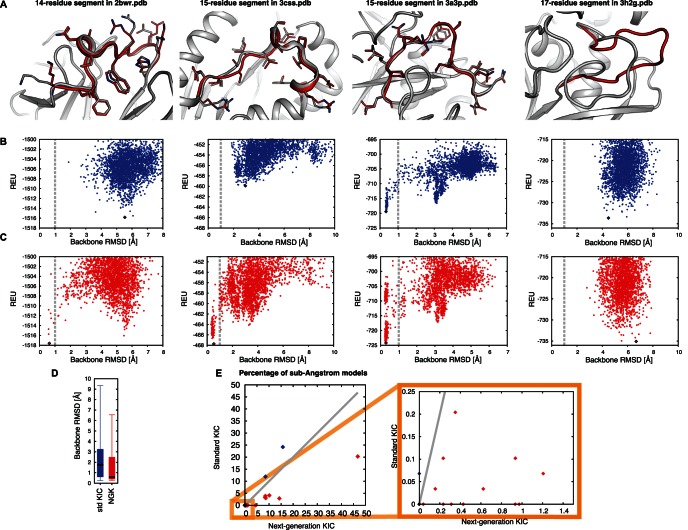
Remodeling Longer Segments. Panels A–C illustrate NGK remodeling results for four selected 14–17 residue segments. (A) Superimposition of the lowest-energy NGK model (red) onto the native structure (gray). Side chains are shown for sub-Angstrom models to illustrate atomic reconstruction accuracy. (B, C) Rosetta energy and backbone RMSD to the native conformation from standard KIC (blue) and NGK (red) simulations generating 2500 models. Diamonds indicate the lowest-energy model. (D) Boxplots with RMSDs of the lowest-scoring models of the KIC (blue) and NGK (red) simulations, respectively. (E) Comparison of the percentage of sub-Angstrom models (%sA) for KIC and NGK across all 28 cases, with specific focus on the cases that could previously only rarely or not at all be reconstructed but are now feasible targets (colors as in Figure 4). Individual RMSDs, %sA and rank for all 28 cases can be found in Table S2.

Given the improvements of NGK over standard KIC, we next sought to apply NGK to a new dataset with increased difficulty in conformational sampling. We chose 28 longer (14–17-residue) segments from an independent dataset that we did not consider during development of NGK (Methods). For 21 cases, or 75% of the test set, NGK was able to sample sub-Angstrom models, six of which could not be sampled by standard KIC (Figure 7, Table S2). For 16 of these 21 cases, sub-Angstrom conformations were correctly identified by the energy function. These correctly identified conformations are very close to the native structure (median backbone RMSD 0.63 Å, Figure 7D) including highly accurate side chain placements (Figure 7A), which is a requirement for backbone remodeling protocols to be used in protein design [1], [16], [37]. The fraction of cases in the benchmark for which NGK successfully sampled sub-Angstrom conformations increases to 87.5% (21/24) if one excludes the four of the 28 segments for which even torsion-restricted sampling cannot generate sub-Angstrom models (for these 4 cases, even dramatically improved sampling methods are likely to fail).

In the long-segment set, five of the seven cases where no sub-Angstrom models were generated as trajectory endpoints appear to be due to sampling deficiencies because the native relaxed conformations had lower energies than the best NGK models. In contrast, four out of the five “identification failures” (sub-Angstrom models sampled, but not lowest-scoring) can be explained by native-relaxed conformations having a worse score than the NGK remodeling results (Table S2).

## Discussion

The sampling improvements in NGK clearly increase the percentage of sub-Angstrom models (16.3%) over that generated by standard KIC (4.3%) and CCD, current state-of-the-art methods for local conformational sampling. In particular, NGK enables reliably sampling sub-Angstrom conformations for six of the 45 benchmark cases in an established 12-residue benchmark set that were previously not found, plus six of 28 cases in a new benchmark set of longer segments. Our results have important implications for more complex protein modeling and design tasks: for example, NGK sampling should help with problems with higher (computational) complexity, such as remodeling multiple or longer segments, by increasing the likelihood of generating native-like conformations. NGK sampling should also lead to better overall predictions in multi-step protocols that are used for challenging design tasks [16], [18], [38], [39], if the lowest-scoring conformation for a given segment can be determined more reliably.

The local conformational sampling benchmarks shown here uses native take-off points for the remodeled loops. For use of KIC or NGK in the context of homology modeling, where exact loop start and end coordinates may not be known, further tests will be necessary to quantify the influence of deviations in take-off points on the accuracy of the modeled conformations. However, a major intended use of NGK is local remodeling during the design of active sites or protein interfaces. In these cases, it may be desirable to only remodel local redesigned regions while leaving the remainder of the known protein scaffold fixed.

Energy function deficiencies revealed by improved sampling methods such as NGK will need to be addressed, and have potentially wide-reaching effects on the overall performance and predictive power of Rosetta. Accurate conformational sampling is essential for developing and assessing improvements of energy functions in high-resolution protein structure modeling and design. For example, modeling the precise geometries of hydrogen bonding interactions requires detailed sampling of both backbone and side chain conformations. Due to its efficient coverage of the local conformational space, NGK provides encouraging avenues and suitable tests for modifications to the energy function.

Despite good overall performance, some cases remain that are not sampled well; remodeling longer segments highlights how such more complex problems are now possible, yet NGK does not generate many sub-Angstrom conformations. To further improve sampling and inspired by the successful application of neighbor-dependent Ramachandran distributions, we intend to extend sequence dependence beyond direct neighbors and extract φ/ψ/ω combinations from fragments. In this fashion, the improved sampling capabilities of NGK and the advantages of fragments-based methods for restricting degrees of freedom can be combined in new, flexible backbone high-resolution modeling [40] and design methods. Further, conformational space annealing, the exchange of low-scoring sub-segments between different models, is an established technique in protein modeling [41], [42] that efficiently exploits information from multiple parallel simulations to identify good conformations. Both techniques may prove especially helpful for remodeling longer segments and other more complex problems, where sampling limitations are most apparent.

## Methods

### Benchmark Datasets

Performance of all individual sampling strategies discussed here as well as their combination was tested on two established 12-residue segment benchmark datasets ([19], [20]), with further curation as described in [4]. For remodeling longer segments, we searched the dataset provided in [43] for instances in which there were at most five residues of the segment to be remodeled within 6 Å of symmetry mates, to minimize the potential influence of crystal contacts on segment conformations. 28 of the 89 segments in the dataset in [43] met this criterion (Table S2).

### Rosetta Kinematic Closure (KIC) Protocol

The Rosetta KIC protocol applies kinematic closure moves (Figure 1A–C) to the selected segment, followed by optimization of the side chains (changing their rotameric conformations) on the segment and within 10 Å of the segment in the full-atom stage. After minimization of the segment’s backbone and side chains within 10 Å, each move is accepted or rejected according to the Monte Carlo Metropolis criterion [4]. After initial closure is achieved, sub-segments of length 3 or more are randomly chosen for further remodeling using the same procedure of sampling non-pivot torsions, closure, side-chain optimization and minimization. Thus, the identity of the pivot residues changes over the course of the protocol. In addition to torsion angle sampling, N-Cα-C-bond angles are sampled from a uniform distribution in the [109.62, 112.1] range, based on bond angle variations observed in crystal structures in the PDB with a resolution of better than 1 Å [4].

Rosetta command line with arguments for standard KIC for PDB 1a8d (loop boundaries can be found in Table S1):

rosetta_source/bin/loopmodel.executable -database rosetta_database/in:file:fullatom -loops:loop_file 1a8d.loop -loops:remodel perturb_kic -loops:refine refine_kic -in:file:native 1a8d_MinPacked.pdb -in:file:s 1a8d_MinPacked.pdb -ex1 -ex2 -extrachi_cutoff 0 -loops:outer_cycles 5 -corrections:score:use_bicubic_interpolation false -kic_bump_overlap_factor 0.36 -legacy_kic false -kic_min_after_repack true.

To ensure that no information from native rotamers was used, all starting structures were repacked using a fixed backbone protocol that performs simultaneous rotamer placement (using only a standard rotamer library expanded by one standard deviation around the side chain χ_1_ and χ_2_ torsion angles [44], [45], [46], but not including native side chain conformations), followed by minimization of the side chain torsion degrees of freedom for each residue [47]:

rosetta_source/bin/fixbb.executable -database rosetta_database/s 1a8d.pdb -ex1 -ex2 -extrachi_cutoff 0 -min_pack -ignore_unrecognized_res -packing:repack_only.

In the KIC protocol, all side chains within 10 Å of the segment are initially discarded and rebuilt after the end of the low-resolution stage, around the conformation at that point in the protocol. As described above, over the course of the high-resolution stage, side chain conformations within 10 Å of the segment are optimized after each new closure step, as described [4].

All data points shown in energy-vs-RMSD plots are trajectory endpoints. Energies are given in Rosetta energy units (REU). For 12-residue segments, 500 models were generated for each benchmark case and tested sampling strategy. For the longer segments, 2500 models were generated per case. The backbone RMSD is calculated by superimposition of the backbone of the native structure and the model excluding the remodeled segment, followed by calculation of the RMSD of the segment backbone’s heavy atoms. Sub-Angstrom conformations are considered “sampled” if there are any models with backbone RMSD <1 Å among the 500 or 2500 generated in the respective simulation. The native conformation is considered “identified” by the Rosetta energy function if the lowest-energy model has a sub-Angstrom RMSD. All simulations were executed with Rosetta SVN revision r51851, and the options implemented here will be available in release 3.5.

### Implementation of Sampling Approaches

#### Diversification strategy: Taboo sampling

For Taboo sampling, the Ramachandran space is split into four sections as described in [22]:

A: φ ≤0 and –130≤ ψ ≤50,

B: φ ≤0 and (ψ<–130 or ψ >50)

E: φ >0 and (ψ<–90 or ψ >90)

G: φ >0 and –90≤ ψ ≤90

For each residue in the segment, the torsion bin as defined above is determined. For each conformation closed by a KIC move, the torsion bin vector (containing a φ/ψ combination for each residue in the remodeled segment) is recorded in the TabooMap. The torsion bin vector always covers the full segment, even if only a subsegment was remodeled in the current trial. Initially, the list of torsion bin vectors to be sampled is solely based on the probability of each torsion bin given the residue type at the respective position. This list has a predetermined size (1000) and is refilled whenever exhausted. For refilling the list, the residue type’s preferences are compared with how often that particular bin has already been sampled at this position, leading to an adjusted frequency of each torsion bin. Vectors matching the TabooMap, i.e., those that have already been tested, are removed from the torsion vector list. To prevent near-deadlock situations, the TabooMap is cleared when 95% of torsion bin space has been covered. For 12 residue loops, this would correspond to 16×10^6^ successful loop closures, which is far beyond the 1200 closure trials performed in our simulations (with 5 outer cycles and 20*12 = 240 inner cycles, Figure 1). Due to the fact that there are up to 2000 closure attempts in each of these trials, the list of torsion bin vectors will be exhausted and refilled several hundred times over the course of a typical trajectory.

In each KIC move, non-pivot torsions are sampled only from the prescribed bin from the next torsion bin vector in the list. To allow for fast sampling within a torsion bin, specific φ/ψ lookup tables are generated for each bin. These tables are subsets of the standard KIC lookup tables but only contain the part of Ramachandran space matching the respective torsion bin. To ensure the Taboo-criterion holds, each torsion bin vector is compared to the current TabooMap directly before it is used for sampling non-pivot torsions. In case this vector has already been tested, it is discarded, and the next vector in the list is considered.

Taboo sampling is activated by adding –loops:taboo_sampling to the Rosetta command line.

#### Intensification strategies: Rama2b and Omega sampling

Neighbor-dependent Ramachandran distributions [24] reflect preferred φ/ψ combinations depending on the local sequence. For kinematic closure with Rama2b sampling, new neighbor-dependent lookup tables for sampling non-pivot φ/ψ combinations are generated. The current Rosetta Rama2b implementation only contains probabilities for the left and right neighbor independently; one side is chosen randomly. For use in combination with Taboo Sampling, the probability of each torsion bin at each position now also needs to consider its direct neighbors. The left and right neighbor are integrated by taking the minimum of both probabilities. As described above, in later iterations of the protocol these probabilities are combined with the frequency recorded in sampling so far.

Rama2b is activated by adding –loops:kic_rama2b to the Rosetta command line.

Omega sampling is based on values observed in high-resolution crystal structures [23]. In the current implementation sampling is independent from the φ/ψ combinations in the remodeled segment. ω is sampled independently at each residue from a Gaussian around the observed mean of 179.1±6.3. For pre-prolines, cis conformations are sampled at a rate of 1/10,000. In the standard KIC implementation [4], all ω angles were assumed to be planar.

Omega sampling is activated by adding –loops:kic_omega_sampling and –allow_omega_move true to the Rosetta command line.

#### Annealing strategies: Ramp repulsive and Ramp rama

Annealing is implemented in the high-resolution stage of the kinematic closure protocol only. The incremental steps are tied to the outer cycles of the protocol (see also Figure 1D), such that a higher number of outer cycles leads to smoother annealing. For *n* outer cycles, the weight is initially reduced to 1/*n* of the full weight as defined by the chosen energy function. Each outer cycle increases the weight by 1/*n*, reaching the full weight as defined in the energy function in the last cycle. In this work we used 5 outer cycles for all KIC and NGK simulations. In the original KIC implementation [4], 3 outer cycles were used without ramping.

Ramp repulsive is activated by adding –loops:ramp_fa_rep to the Rosetta command line, Ramp rama by adding –loops:ramp_rama.

Note that, when using Ramp rama together with Rama2b, the weight of the Rama2b term is ramped analogously to the annealing scheme for rama, while the weight for the standard rama term is set to 0.

### Next-generation KIC (NGK)

NGK is a combination of Rama2b, Omega sampling, Ramp repulsive and Ramp rama and is thus activated by adding the following flags to the standard KIC command line:

–loops:kic_rama2b –loops:kic_omega_sampling –allow_omega_move true –loops:ramp_fa_rep –loops:ramp_rama.

The Protocol Capture accompanying this manuscript contains the command line options for running NGK, as well as example input files (Dataset S1).

Synergy is assessed by comparing the expected to the observed median value of the fraction of sub-Angstrom models (m%sA). The expected m%sA is calculated by adding the contributions from each individual improvement (m%sA(respective method) - m%sA(standard KIC)) to the m%sA of standard KIC, plus the sum of their standard deviations.

### Torsion-restricted Sampling

Torsion-restricted sampling utilizes the same torsion-bin-specific φ/ψ lookup tables as Taboo sampling. However, unlike for Taboo sampling, the torsion bin vector is constant over the course of the trajectory, so that φ/ψ torsions are sampled in every step from their respective native torsion bin. Matching lowercase identifiers are used to denote cis ω angles, so that segments with cis-prolines will always combine the bin-dependent φ/ψ torsion angle values with an ω of 0. One possible caveat with torsion-restricted sampling is that it is “blind” to alternative funnels if those are in conformations with a torsion bin vector that differs from the native one. Thus, while torsion-restricted sampling reveals whether intensive sampling yields near-native conformations, it cannot determine whether this is the lowest-scoring funnel in the energy landscape.

Torsion-restricted sampling with automatic derivation of the appropriate torsion bin vector for the native conformation is enabled by adding -derive_torsion_string_from_native_pose -kic_omega_sampling -allow_omega_move true to the standard KIC command line.

### Fragment Insertion Followed by Cyclic Coordinate Descent (CCD)

Local conformational sampling by fragment insertion and CCD was carried out as described [34], but with 5 outer cycles for comparability to the KIC and NGK simulations in this work as opposed to the 3 default outer cycles:

rosetta_source/bin/loopmodel.executable –database rosetta_database/s 1a8d_MinPacked.pdb -in:file:fullatom -loops:loop_file 1a8d.loop -loops:remodel quick_ccd -loops:refine refine_ccd -in:file:native 1a8d_MinPacked.pdb -loops:frag_sizes 9 3 1 –loops:frag_files 1a8dA.200.9mers 1a8dA.200.3mers none -ex1 -ex2 -extrachi_cutoff 0 -loops:outer_cycles 5.

Fragments were generated as described in [35].

### Locally Relaxed Native-like Conformations

To assess whether sampling and identification failures are more likely to be problems with sampling or with scoring, we applied Rosetta’s FastRelax protocol [28] within the RosettaScripts framework [48] on the starting structures used for KIC and NGK simulations. The FastRelax protocol applies iterative repacking and minimization on the segment’s backbone φ,ψ and ω torsion degrees of freedom and on the side chain torsions in the entire structure, while ramping the weight of the repulsive component of the Lennard-Jones potential, applying uniform constraints to the native coordinates. Five such iterations are carried out to generate low-scoring native-like models.

rosetta_source/bin/rosetta_scripts.executable -database rosetta_database/ex1 -ex2 -extrachi_cutoff 0 -constrain_relax_to_start_coords -parser:protocol fastrelax_1a8d_segment.xml -s 1a8d_MinPacked.pdb.

With fastrelax_1a8d_segment.xml as follows:

<ROSETTASCRIPTS>.

<TASKOPERATIONS>.

<InitializeFromCommandline name = read_cmdline/>.

</TASKOPERATIONS>.

<MOVERS>.

<FastRelax name = fastrelax_loop task_operations = read_cmdline >.

<MoveMap name = mm>.

<Chain number = 1 chi = 1 bb = 0/>.

<Span begin = 155 end = 166 chi = 1 bb = 1/> remodeled segment.

</MoveMap>.

</FastRelax>.

</MOVERS>.

<PROTOCOLS>.

<Add mover = fastrelax_loop/>.

</PROTOCOLS>.

</ROSETTASCRIPTS>.

## Supporting Information

Figure S1 **Clustering of Sampled Conformations Identifies sub-Angstrom Conformations Contained in the Largest Cluster.** Applying the Rosetta clustering application [25] with a cluster radius of 1 Å on the remodeled segment yields four clusters with at least 30 models. The largest of those clusters (red) contains many sub-Angstrom conformations, which were considerably enriched by Rama2b sampling. (A) Energy-vs-RMSD plot from Rama2b sampling as in Fig. 2C, colored by cluster. (B) The lowest-scoring model from each cluster. Colors as in (A).
(PDF)

Figure S2 **RMSD and percentage of sub-Angstrom conformations with fixed Cα bond angles.** (A) RMSDs observed for standard KIC (blue, as in Fig. 3), KIC with fixed Cα bond angles (green), NGK (red, as in Fig. 3) and NGK with fixed bond angles (orange). Boxplots show minimum and maximum among the lowest-scoring RMSDs across the benchmark set (error bars), the 25^th^ and 75^th^ percentile (box boundaries) as well as the median (thick line). (B) Barplots of median percentage of sub-Angstrom models. Colors as in (A).
(PDF)

Table S1 **RMSDs for the 12-residue Benchmark Set and Analysis of Scoring/Sampling Failures.** PDB identifiers, loop boundaries, RMSD of the lowest-energy model for standard KIC, CCD and NGK, respectively, bond angle outliers as determined by MolProbity [30] and, in the case of sampling failures or identification failures, an indication for whether the native like-relaxed model scores lower than the lowest-energy NGK model generated in our simulations (“energy gap”).
(XLSX)

Table S2 **Details for Long Remodeled Segments.** PDB identifiers, loop boundaries, percentage of sub-Angstrom models (%sA) and rank of the lowest-energy model for the 28 long segments remodeled in this work for standard KIC, torsion-restricted sampling and next-generation KIC (NGK) simulations. For KIC and NGK, the RMSD of the lowest-scoring model is also provided. 2500 models were generated with standard KIC and NGK, 500 with torsion-restricted sampling.
(XLSX)

Dataset S1 **Protocol Capture.** The protocol capture describes all command line options required for running NGK, as well as example input and output files.
(GZ) Click here for additional data file.
