# Supplementary figures and images for: Improvements to Robotics-Inspired Conformational Sampling in Rosetta
Source: PLoS One. 2013 May 21;8(5):e63090. doi: 10.1371/journal.pone.0063090 (PMC3660577; doi:10.1371/journal.pone.0063090)

**A****Clustered models from Rama2b sampling (1m3s.pdb)**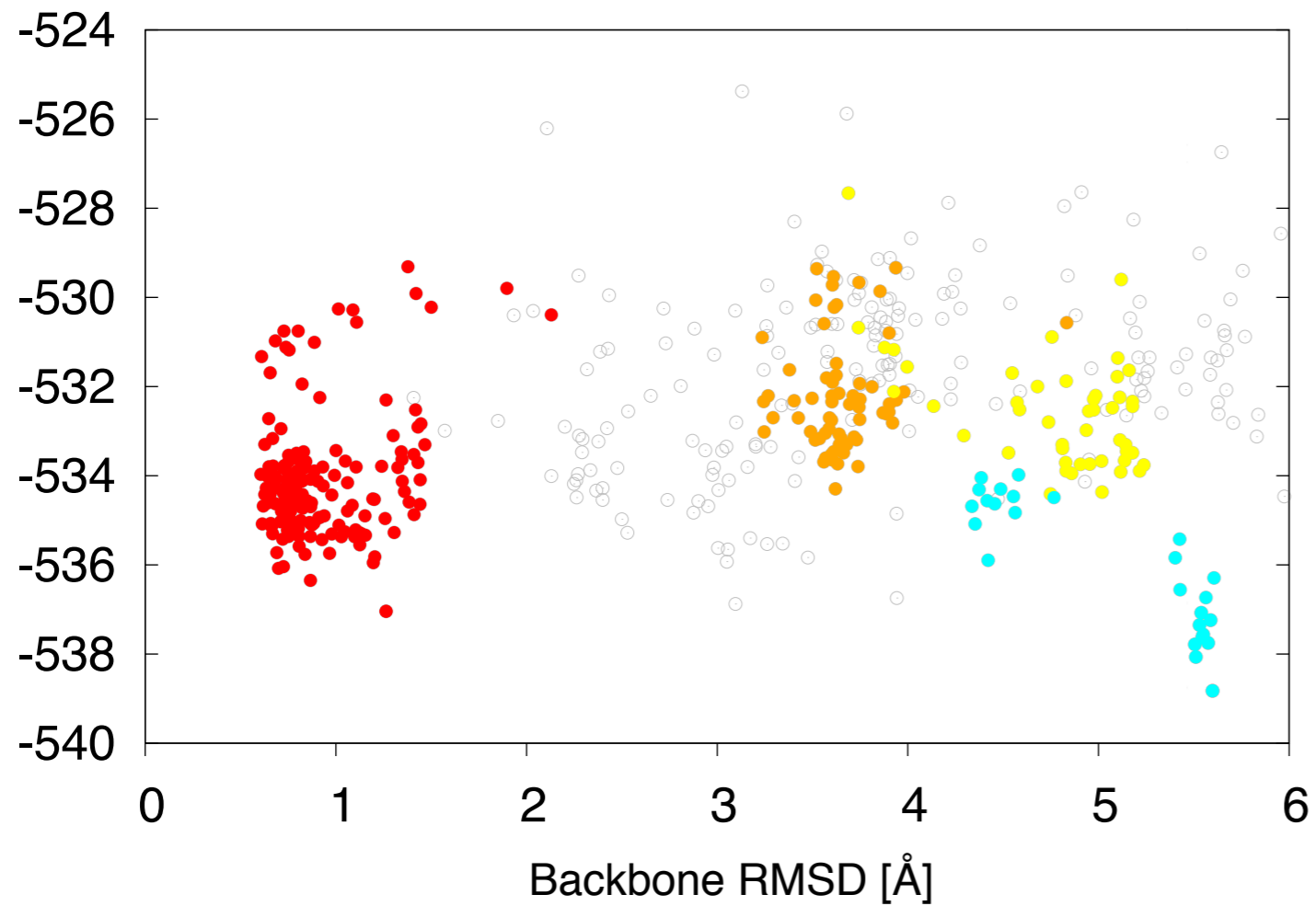**B**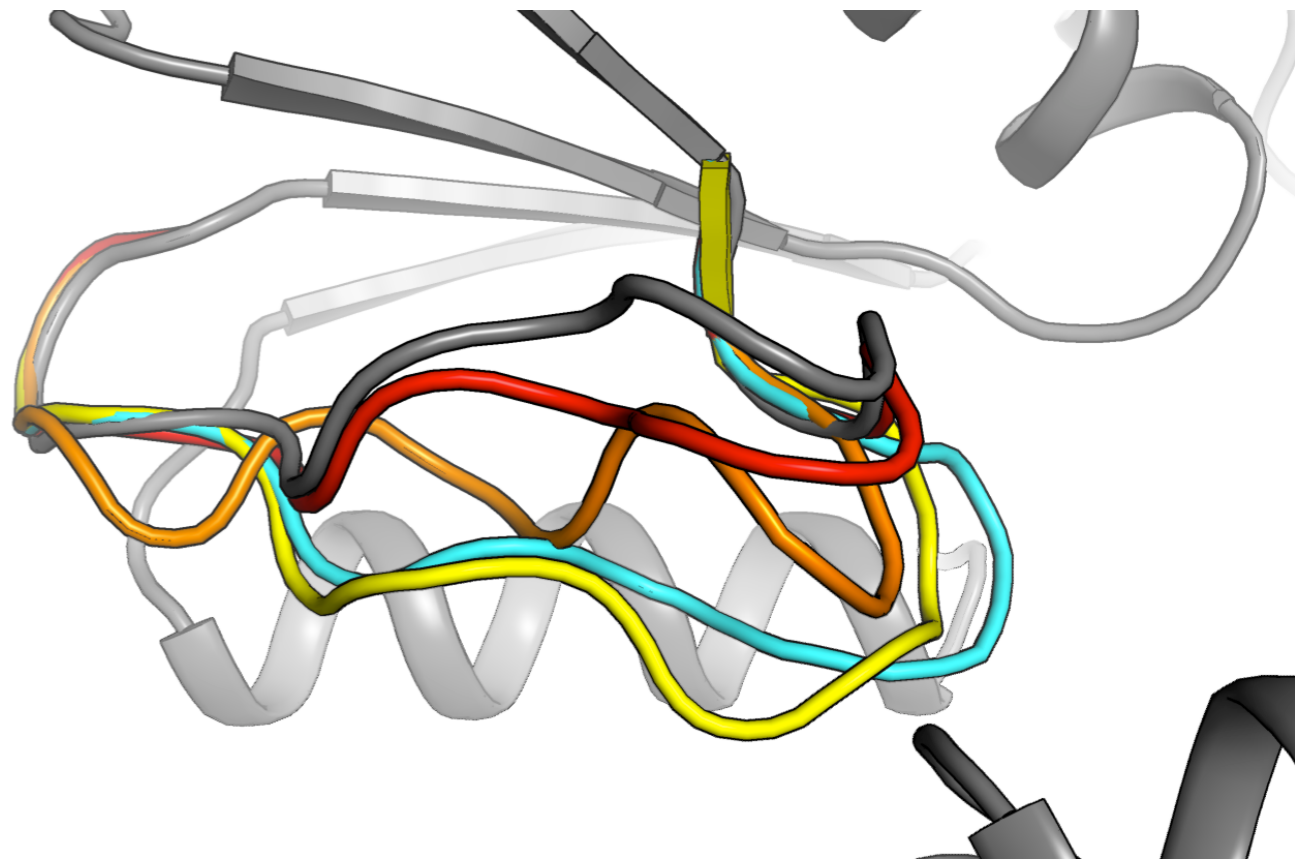

Supplement: Figure S1 — Clustering of Sampled Conformations Identifies sub-Angstrom Conformations Contained in the Largest Cluster. Applying the Rosetta clustering application [25] with a cluster radius of 1 Å on the remodeled segment yields four clusters with at least 30 models. The largest of those clusters (red) contains many sub-Angstrom conformations, which were considerably enriched by Rama2b sampling. (A) Energy-vs-RMSD plot from Rama2b sampling as in Fig. 2C, colored by cluster. (B) The lowest-scoring model from each cluster. Colors as in (A). (PDF) [file pone.0063090.s001.pdf]

**A** Backbone RMSD [ $\text{\AA}$ ]

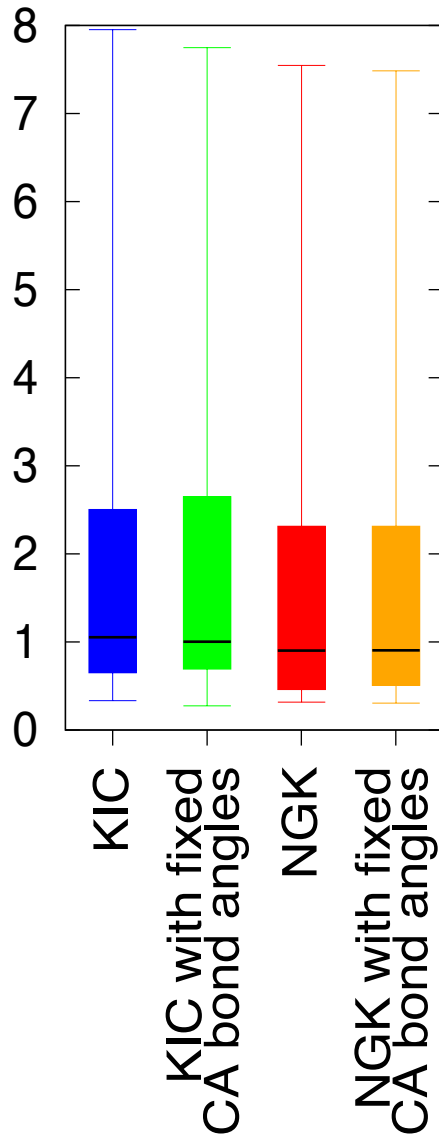

**B** median % sub-Angstrom models

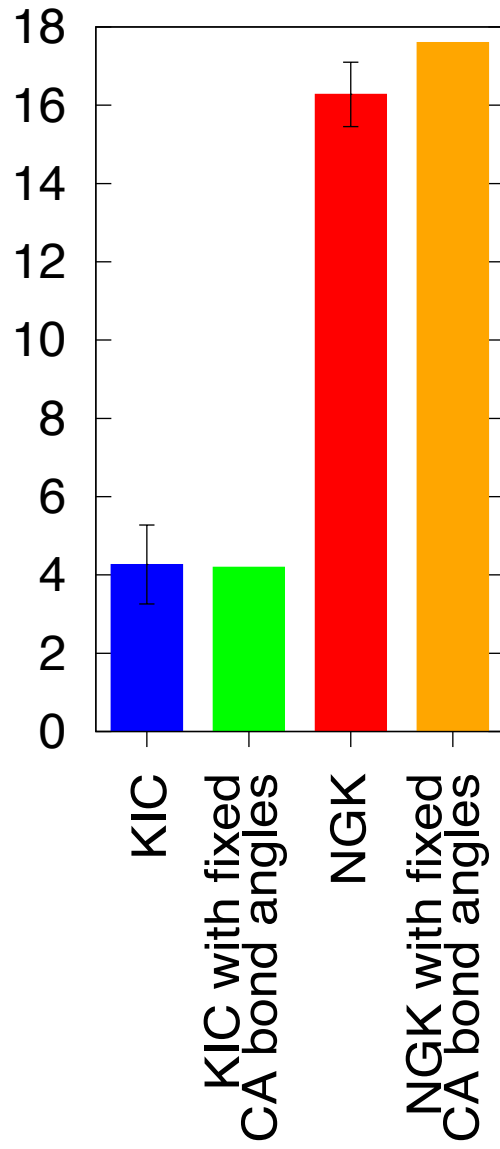

Supplement: Figure S2 — RMSD and percentage of sub-Angstrom conformations with fixed Cα bond angles. (A) RMSDs observed for standard KIC (blue, as in Fig. 3), KIC with fixed Cα bond angles (green), NGK (red, as in Fig. 3) and NGK with fixed bond angles (orange). Boxplots show minimum and maximum among the lowest-scoring RMSDs across the benchmark set (error bars), the 25th and 75th percentile (box boundaries) as well as the median (thick line). (B) Barplots of median percentage of sub-Angstrom models. Colors as in (A). (PDF) [file pone.0063090.s002.pdf]
